# Supplementary material for: Methods to Stimulate Sporulation and Freeze-Drying Strategies for the Conservation of Diplodia mutila, Diplodia seriata, Lasiodiplodia theobromae, and Neofusicoccum arbuti Isolated from Apple Trees with Canker and Dieback Symptoms
Source: J Fungi (Basel). 2025 Aug 29;11(9):640. doi: 10.3390/jof11090640 (PMC12470272; doi:10.3390/jof11090640)
Supplement: Supplementary file 1 [file jof-11-00640-s001.zip › jof-3810709-supplementary.pdf]

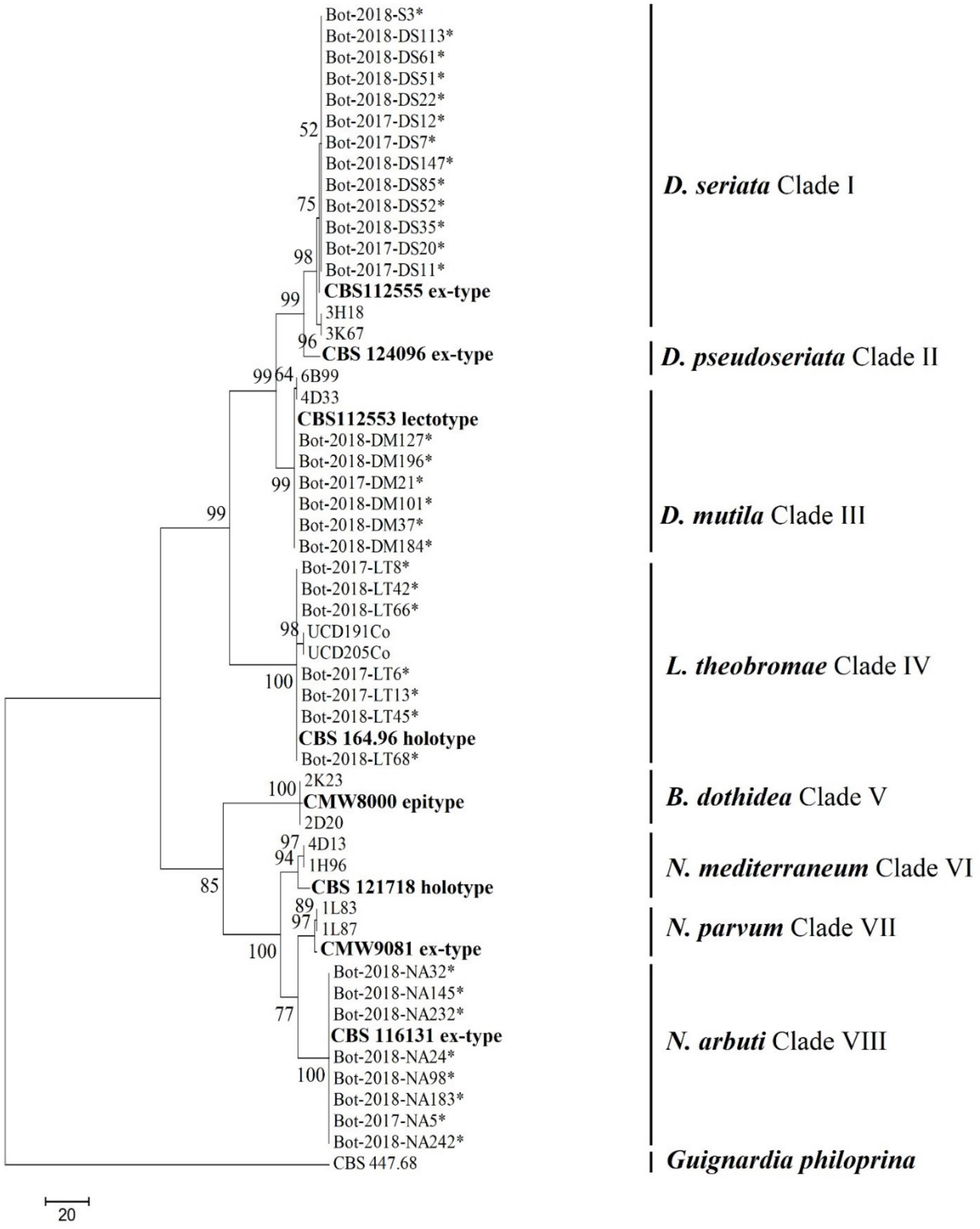

Supplementary Figure S1. One of the seven most parsimonious trees inferred from maximum parsimony phylogeny of *Botryosphaeriaceae* species according to concatenated alignments of sequences of the internal transcribed spacer (*ITS*), portion of translation elongation factor 1-α (*tef1*) and portion of the beta tubulin (*tub2*) gene regions. Bootstrap support values from 1,000 replicates >50% are reported at the nodes. The values were obtained with MEGA version 7.0. *Botryosphaeriaceae* isolates collected in this study are indicated with an asterisk. The ex-type/ex-epitype/ex-holotype strains are indicated in bold.

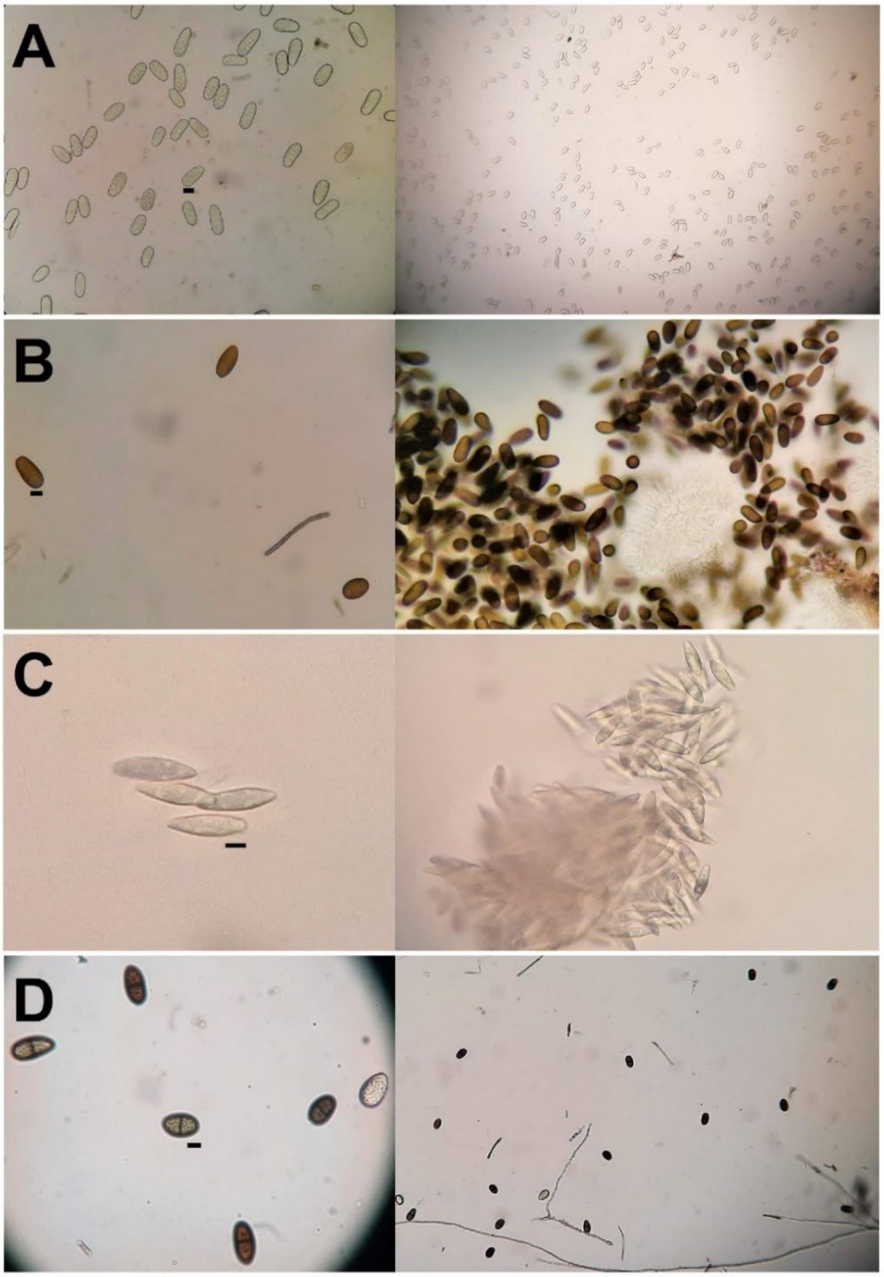

Supplementary Figure S2. Production of conidia. Agar-water medium (W-A) at 2% with the addition of pine needles in petri dishes (P.D), development temperature 24 ºC and 15 days of exposure to light (UV) λ = 350 nm under a dark camera. Microscope (Nikon), 100 and 40× magnifications. **(A)** *Diplodia mutila* (Bot-2017-DM21), **(B)** *Diplodia seriata* (Bot-2018-S3), **(C)** *Neofusicoccum arbuti* (Bot-2018-NA32) and **(D)** *Lasiodiplodia theobromae* (Bot -2018-LT45). Scale bar = 10 μm.

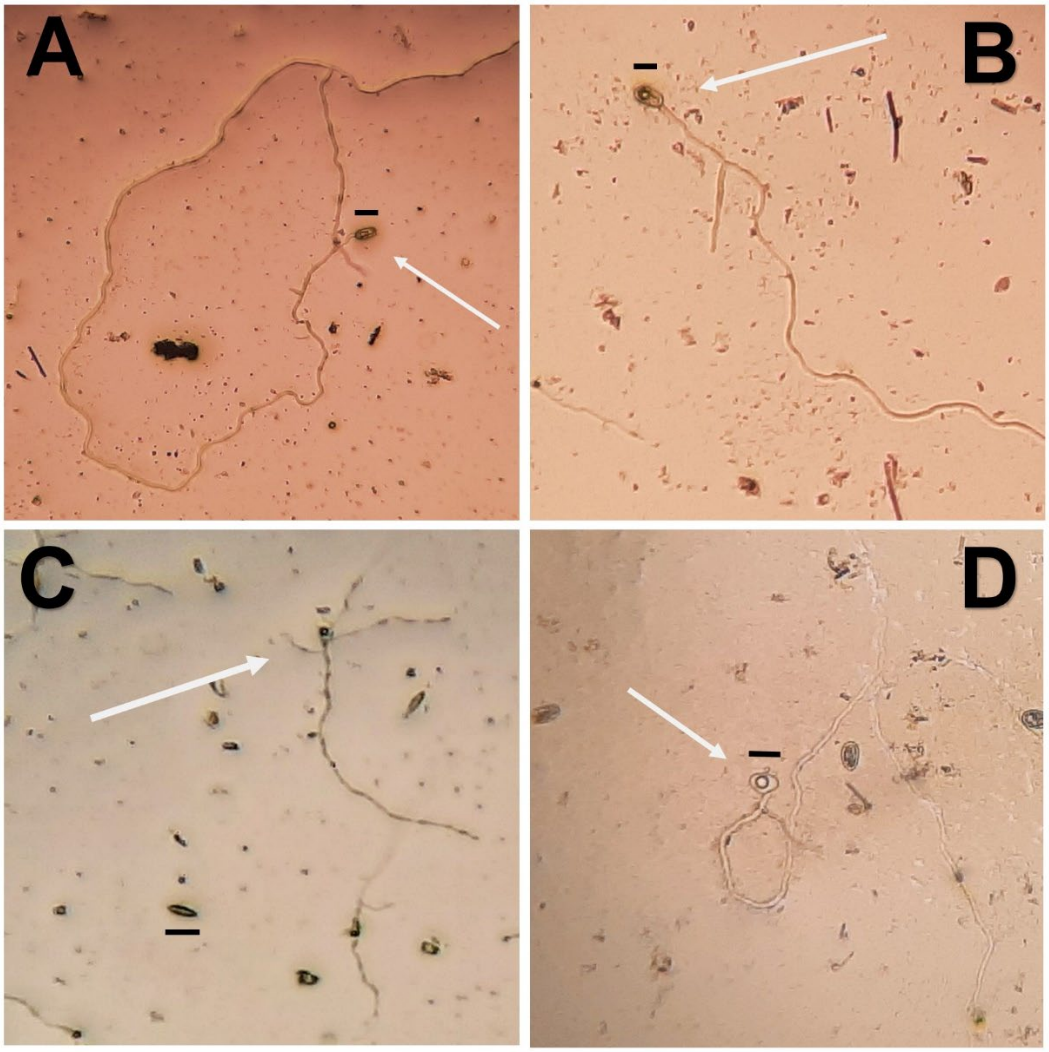

Supplementary Figure S3. Conidial germination after 365 days of the freeze-drying process. Conidial viability was assessed in 0.1% PDA medium plus 0.01% tetracycline hydrochloride incubated at 24 ± 2ºC after 24 hours. Arrows indicate conidia, germ tube development, and viability of: **(A)** *Diplodia mutila* (Bot-2017-DM21), **(B)** *Diplodia seriata* (Bot-2018-S3), **(C)** *Neofusicoccum arbuti* (Bot-2018-NA32) and **(D)** *Lasiodiplodia theobromae* (Bot-2018-LT45). Microscope (Nikon), magnification 40×. Scale bar = 20 μm.

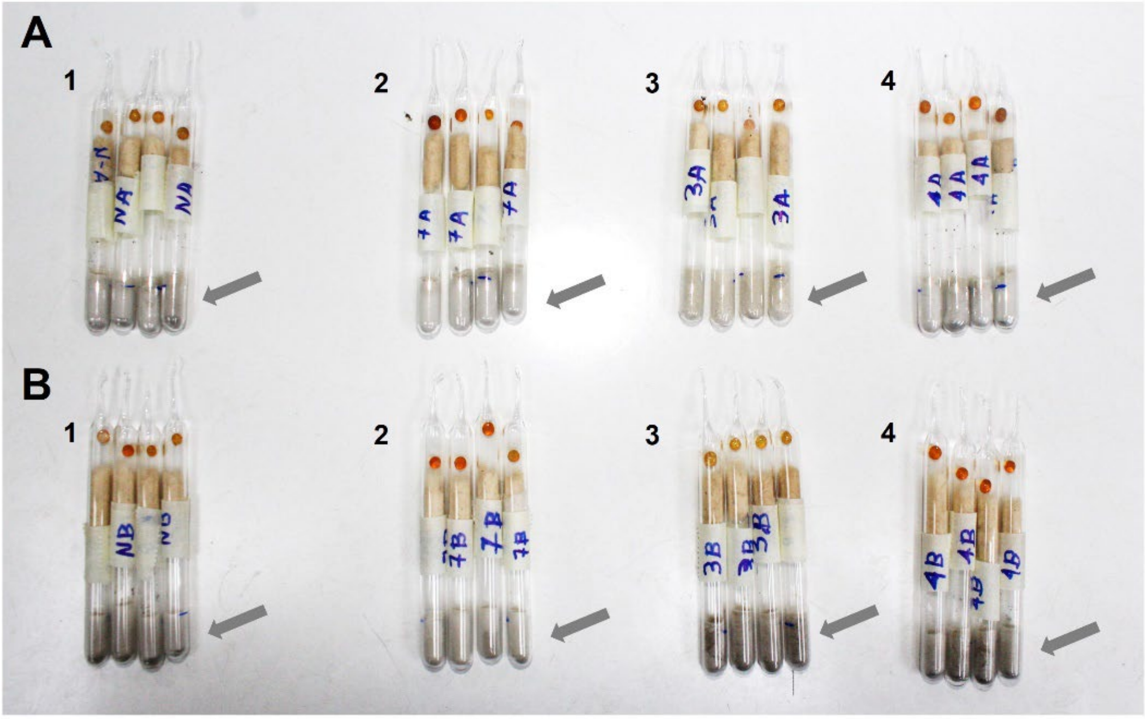

Supplementary Figure S4. Glass ampoules (G.A.) vacuum sealed containing freeze-dried *Botryosphaeriaceae* spp. The final result of the freeze-drying process is shown in two groups of glass ampoules: **(A)** group consisting of 20% smp. (skimmed milk powder) protective medium and **(B)** group consisting of 20% smp. protective medium plus 5% inositol. The number indicates the species: **(1)** *Neofusicoccum arbuti* (Bot-2018-NA32), **(2)** *Diplodia mutila* (Bot-2017-DM21), **(3)** *Lasiodiplodia theobromae* (Bot-2018-LT45) and **(4)** *Diplodia seriata* (Bot-2018-S3). The arrows indicate the freeze-dried protective medium at the bottom of the glass ampoule. Canon D600 camera.

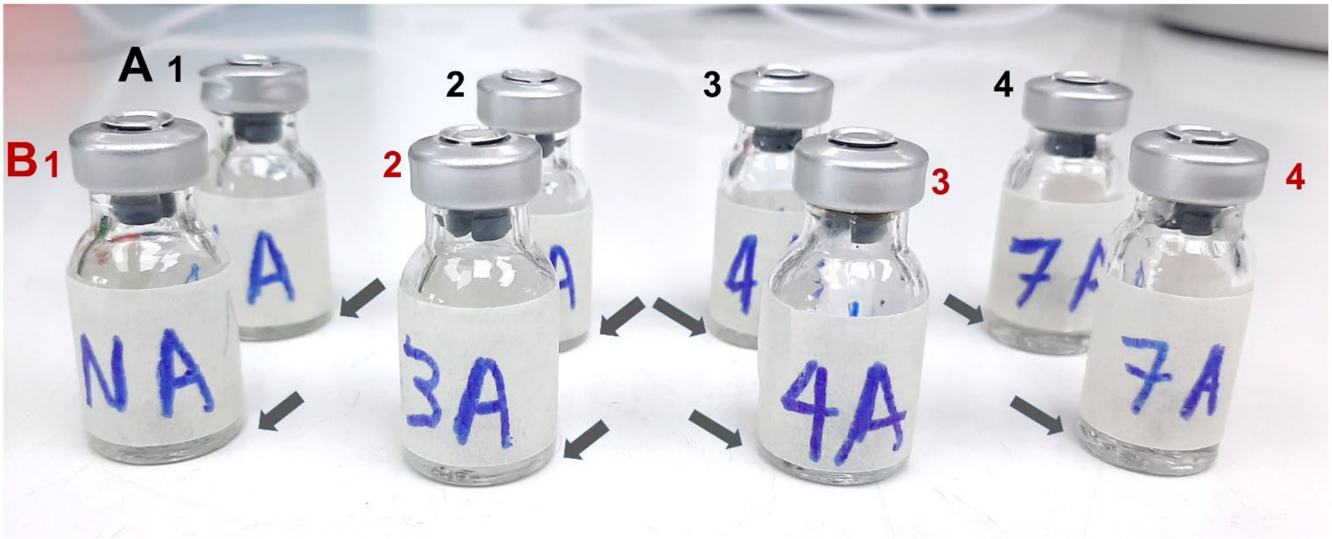

Supplementary Figure S5. Glass vials (G.V.) containing lyophilized *Botryosphaeriaceae* spp. The final result of the lyophilization process is shown in two groups of glass vials: **(A)** group composed of 20% smp. (skimmed milk powder) protective medium and **(B)** group composed of 20% smp. protetive medium plus 5% inositol. The number indicates the species: **(1)** *Neofusicoccum arbuti* (Bot-2018-NA32), **(2)** *Lasiodiplodia theobromae* (Bot-2018-LT45), **(3)** *Diplodia seriata* (Bot-2018-S3) and **(4)** *Diplodia mutila* (Bot-2017-DM21). The arrows indicate the lyophilized protective medium at the bottom of each glass vial. Canon D600 camera.

Supplementary Table S1. Primer sequences used for the amplification of *ITS*, translation elongation factor 1-α (*tef1-α*), and β-tubulin (*tub2*) genes in *Diplodia mutila* (Bot-2017-DM21), *Diplodia seriata* (Bot-2018-S3), *Neofusicoccum arbuti* (Bot-2018-NA32) and *Lasiodiplodia theobromae* (Bot-2018-LT45).

Gen	Primers (Forward/Reverse)	Sequence (5′–3′)	Reference
ITS (rDNA)	ITS1 / ITS4	TCCGTAGGTGAACCTGCGG / TCCTCCGCTTATTGATATGC	[46]
tef1-α (Translation elongation factor 1-α)	EF1-728F / EF1-986R	CATCGAGAAGTTCGAGAAGG / TACTTGAAGGAACCCTTACC	[47]
tub2 (β-tubulin)	Bt2a / Bt2b	GGTAACCAAATCGGTGCTGCTTTC / ACCCTCAGTGTAGTGACCCTTGGC	[48]

Supplementary Table S2. Culture media and stimulation conditions for conidia production in *Botryosphaeriaceae* spp. isolated from apple twigs with dieback and canker symptoms.

**Medium culture** [x]	**Mean total** [y]	**Contai-ner** [z]	**Days/ Temperature °C**	**Days/ temperature °C UV(λ = 350 nm)**	**(n) Mean conidia** [w]			
					D. mutila	***D. seriata***	***N. arbuti***	***L. theobromae***
PDA	0 e	P.D.	30/24	15/24	-	-	-	-
PDA	0 e	P.D.	30/24	-	-	-	-	-
PDA	0 e	P.D.	30/24 + 30/5	15/24	-	-	-	-
PDA	0 e	P.D.	30/24 + 30/5	-	-	-	-	-
Agar-water	0 e	P.D.	30/24	15/24	-	-	-	-
Agar-water	0 e	P.D.	30/24	-	-	-	-	-
PDA	0 e	I.T.T	30/24	15/24	-	-	-	-
PDA	0 e	I.T.T	30/24	-	-	-	-	-
Agar-water	21.0 d	I.T.T	30/24	15/24	-	-	-	84.2
Agar-water	0 e	I.T.T	30/24	-	-	-	-	-
Agar-water+pine needles	537.9 b	P.D.	30/24	15/24	552	671.2	445.2	483.2
Agar-water+pine needles	0 e	P.D.	30/24	-	-	-	-	-
PDA + pine needles	48.4 c	P.D.	30/24	15/24	54	75.7	46	18
PDA + pine needles	0 e	P.D.	30/24	-	-	-	-	-
PDA + pine needles	45.6 c	P.D.	30/24 + 30/5	15/24	59.7	60	51.2	11.7
PDA + pine needles	0 e	P.D.	30/24 + 30/5	-	-	-	-	-
PDA	0 e	F.	30/24	15/24	-	-	-	-
PDA	0 e	F.	30/24 + 30/5	15/24	-	-	-	-
Agar-water + vine chips	542.1b	P.D.	30/24	15/24	594.7	612.2	490	471.5
Agar-water + vine chips	0 e	P.D.	30/24	-	-	-	-	-
Agar-water+apple chips	573.7 a	P.D.	30/24	15/24	600.7	602.2	601.5	490.7
Agar-water+apple chips	0 e	P.D.	30/24	-	-	-	-	-

[w] Mean values of four replicates per treatment of the number of conidia produced by *Diplodia mutila* (Bot-2017- DM2), *Diplodia seriata* (Bot-2018-S3), *Neofusicoccum arbuti* (Bot-2018-NA32) and *Lasiodiplodia theobromae* (Bot-2018-LT45) (RGM 3136).

[x] Culture media and their variations evaluated.

[y] Total mean values of the number of conidia produced by the four *Botryosphaeriaceae* spp. evaluated in each treatment. Values with the same letters are not significantly different according to the *Tukey* test (P-value <0.0001).

[z] Culture medium container type: P.D = Petri dishes, I.T.T.= inclined test tubes and F.= 250 ml flask.

Supplementary Table S3a. Viability and survival of *Diplodia mutila* (Bot-2017-DM21), *Diplodia seriata* (Bot-2018-S3), *Neofusicoccum arbuti* (Bot-2018-NA32) and *Lasiodiplodia theobromae* (Bot-2018-LT45) after the freeze-drying process over time.

Insolated	Days	Container[b]	Protective medium[c]		CFU[a]				% RV[d]
					10^{-4}	10^{-5}	10^{-6}	10^{-7}	
D. mutila	1	G.V.	Smp	c	456	46	4	-	-
		G.V.	Smp + inositol	b	493	50	4	-	-
		G.A.	Smp	a	501	51	5	-	-
		G.A.	Smp + inositol	a	505	51	6	-	-
	90	G.V.	Smp	d	448	45	4	-	1.8
		G.V.	Smp + inositol	b	489	49	4	-	0.8
		G.A.	Smp	a	498	50	5	-	0.6
		G.A.	Smp + inositol	a	505	50	6	-	0.0
	180	G.V.	Smp	d	443	44	4	-	2.9
		G.V.	Smp + inositol	b	485	49	4	-	1.6
		G.A.	Smp	a	496	50	5	-	1.0
		G.A.	Smp + inositol	a	505	51	6	-	0.0
	365	G.V.	Smp	e	429	43	4	-	5.9
		G.V.	Smp + inositol	b	475	48	4	-	3.7
		G.A.	Smp	a	486	49	5	-	3.0
		G.A.	Smp + inositol	a	491	50	6	-	2.8
D. seriata	1	G.V.	Smp	c	561	56	5	-	-
		G.V.	Smp + inositol	b	583	58	6	-	-
		G.A.	Smp	a	609	60	6	-	-
		G.A.	Smp + inositol	a	605	65	7	-	-
	90	G.V.	Smp	d	548	55	5	-	2.3
		G.V.	Smp + inositol	b	575	58	6	-	1.4
		G.A.	Smp	a	607	61	6	-	0.3
		G.A.	Smp + inositol	a	600	61	7	-	0.8
	180	G.V.	Smp	d	544	55	5	-	3.0
		G.V.	Smp + inositol	b	569	56	6	-	2.4
		G.A.	Smp	a	595	59	6	-	2.3
		G.A.	Smp + inositol	a	597	59	7	-	1.3
	365	G.V.	Smp	e	530	54	4	-	5.5
		G.V.	Smp + inositol	b	559	55	6	-	4.1
		G.A.	Smp	a	591	60	6	-	3.0
		G.A.	Smp + inositol	a	590	60	7	-	2.5

[a] Number of colony forming units **(CFU)**, counted in four serial dilution values.

[b] Type of container used for the freeze-drying process. **(G.V.)** glass vials and **(G.A.)** glass ampoules.

[c] Protective medium used for the freeze-drying process. (smp) 20% skimmed milk powder. (smp + inositol) 20% evaporated skimmed milk plus the addition of 5% inositol. The same letters in each species are not significantly different according to the *Scheffé* test (p value <0.0001).

[d] Percentage reduction **(RV)** in viability after the freeze-drying process, over time.

Supplementary Table S3b. Viability and survival of *Diplodia mutila* (Bot-2017-DM21), *Diplodia seriata* (Bot-2018-S3), *Neofusicoccum arbuti* (Bot-2018-NA32) and *Lasiodiplodia theobromae* (Bot-2018-LT45) after the freeze-drying process over time.

Insolated	Days	Container[b]	Protective medium[c]		CFU[a]				% RV[d]
					10^{-4}	10^{-5}	10^{-6}	10^{-7}	
N. arbuti	1	G.V.	Smp	c	309	32	3	-	-
		G.V.	Smp + inositol	b	324	33	3	-	-
		G.A.	Smp	a	343	36	3	-	-
		G.A.	Smp + inositol	a	368	38	4	-	-
	90	G.V.	Smp	d	307	31	3	-	0.8
		G.V.	Smp + inositol	b	322	32	3	-	0.6
		G.A.	Smp	a	341	35	3	-	0.5
		G.A.	Smp + inositol	a	367	37	4	-	0.4
	180	G.V.	Smp	d	302	31	3	-	2.4
		G.V.	Smp + inositol	b	318	32	3	-	1.9
		G.A.	Smp	a	337	34	3	-	1.7
		G.A.	Smp + inositol	a	363	36	4	-	1.4
	365	G.V.	Smp	e	291	30	3	-	5.9
		G.V.	Smp + inositol	b	307	31	3	-	5.2
		G.A.	Smp	a	327	32	3	-	4.6
		G.A.	Smp + inositol	a	353	36	4	-	4.2
L. theobromae	1	G.V.	Smp	c	401	39	4	-	-
		G.V.	Smp + inositol	b	415	41	4	-	-
		G.A.	Smp	a	428	43	4	-	-
		G.A.	Smp + inositol	a	477	48	5	-	-
	90	G.V.	Smp	d	399	41	4	-	0.5
		G.V.	Smp + inositol	b	413	44	4	-	0.5
		G.A.	Smp	a	427	44	4	-	0.2
		G.A.	Smp + inositol	a	476	48	5	-	0.2
	180	G.V.	Smp	d	397	38	4	-	1.0
		G.V.	Smp + inositol	b	410	40	4	-	1.2
		G.A.	Smp	a	425	43	4	-	0.7
		G.A.	Smp + inositol	a	474	49	5	-	0.6
	365	G.V.	Smp	e	387	38	4	-	3.5
		G.V.	Smp + inositol	b	402	41	4	-	3.1
		G.A.	Smp	a	416	43	4	-	2.8
		G.A.	Smp + inositol	a	465	47	6	-	2.5

[a] Number of colony forming units **(CFU)**, counted in four serial dilution values.

[b] Type of container used for the freeze-drying process. **(G.V.)** glass vials and **(G.A.)** glass ampoules.

[c] Protective medium used for the freeze-drying process. (smp) 20% skimmed milk powder. (smp + inositol) 20% evaporated skimmed milk plus the addition of 5% inositol. The same letters in each species are not significantly different according to the *Scheffé* test (*p* value <0.0001).

[d] Percentage reduction **(RV)** in viability after the freeze-drying process, over time.
